# Do GWAS-Identified Risk Variants for Chronic Lymphocytic Leukemia Influence Overall Patient Survival and Disease Progression?

**DOI:** 10.3390/ijms24098005

**Published:** 2023-04-28

**Authors:** Antonio José Cabrera-Serrano, José Manuel Sánchez-Maldonado, Rob ter Horst, Angelica Macauda, Paloma García-Martín, Yolanda Benavente, Stefano Landi, Alyssa Clay-Gilmour, Yasmeen Niazi, Blanca Espinet, Juan José Rodríguez-Sevilla, Eva María Pérez, Rossana Maffei, Gonzalo Blanco, Matteo Giaccherini, James R. Cerhan, Roberto Marasca, Miguel Ángel López-Nevot, Tzu Chen-Liang, Hauke Thomsen, Irene Gámez, Daniele Campa, Víctor Moreno, Silvia de Sanjosé, Rafael Marcos-Gragera, María García-Álvarez, Trinidad Dierssen-Sotos, Andrés Jerez, Aleksandra Butrym, Aaron D. Norman, Mario Luppi, Susan L. Slager, Kari Hemminki, Yang Li, Sonja I. Berndt, Delphine Casabonne, Miguel Alcoceba, Anna Puiggros, Mihai G. Netea, Asta Försti, Federico Canzian, Juan Sainz

**Affiliations:** 1 Genomic Oncology Area, GENYO, Centre for Genomics and Oncological Research: Pfizer/University of Granada/Andalusian Regional Government, PTS, 18016 Granada, Spain; antonio.cabrera@genyo.es (A.J.C.-S.); josemanuel.sanchez@genyo.es (J.M.S.-M.); 2 Instituto de Investigación Biosanitaria IBs.Granada, 18012 Granada, Spain; 3 CeMM Research Center for Molecular Medicine of the Austrian Academy of Sciences, 1090 Vienna, Austria; rob.terhorst@radboudumc.nl; 4 Genomic Epidemiology Group, German Cancer Research Center (DKFZ), 69120 Heidelberg, Germany; angelicamacauda@gmail.com (A.M.); f.canzian@dkfz.de (F.C.); 5 Hospital Campus de la Salud, PTS, 18016 Granada, Spain; palomagrm@hotmail.com (P.G.-M.); evam.perez.sspa@juntadeandalucia.es (E.M.P.); 6 Catalan Institute of Oncology, Bellvitge Biomedical Research Institute (IDIBELL), 08908 Barcelona, Spain; ybenavente@iconcologia.net (Y.B.); v.moreno@iconcologia.net (V.M.); s.sanjose@iconcologia.net (S.d.S.); dcasabonne@iconcologia.net (D.C.); 7 Consortium for Biomedical Research in Epidemiology and Public Health (CIBERESP), University of Barcelona, 08908 Barcelona, Spain; rmarcos@iconcologia.net (R.M.-G.); trinidad.dierssen@unican.es (T.D.-S.); 8 CIBER Epidemiología y Salud Pública (CIBERESP), 28029 Madrid, Spain; 9 Department of Biology, University of Pisa, 56126 Pisa, Italy; stefano.landi@unipi.it (S.L.); matteo.giaccherini@phd.unipi.it (M.G.); daniele.campa@unipi.it (D.C.); 10 Department of Epidemiology & Biostatistics, Arnold School of Public Health, University of South Carolina, Greenville, SC 29208, USA; claygila@mailbox.sc.edu; 11 Division of Pediatric Neurooncology, German Cancer Research Center (DKFZ), German Cancer Consortium (DKTK), 69120 Heidelberg, Germany; y.niazi@dkfz.de (Y.N.); a.foersti@kitz-heidelberg.de (A.F.); 12 Hopp Children’s Cancer Center (KiTZ), 69120 Heidelberg, Germany; 13 Molecular Cytogenetics Laboratory, Pathology Department, Hospital del Mar, 08003 Barcelona, Spain; bespinet@parcdesalutmar.cat (B.E.); gblancoares@northwell.edu (G.B.); apuiggros@psmar.cat (A.P.); 14 Translational Research on Hematological Neoplasms Group, Cancer Research Program, Institut Hospital del Mar d’Investigacions Mèdiques (IMIM), 08003 Barcelona, Spain; 15 Department of Leukemia, The University of Texas MD Anderson Cancer Center, Houston, TX 77030, USA; jrodsevilla@gmail.com; 16 Department of Medical and Surgical Sciences, University of Modena and Reggio Emilia, AOU Policlinico, 41124 Modena, Italy; maffei.rossana@aou.mo.it (R.M.); roberto.marasca@unimore.it (R.M.); mario.luppi@unimore.it (M.L.); 17 Department of Quantitative Health Sciences, Mayo Clinic, Rochester, MN 55905, USA; buehler@mayo.edu (J.R.C.); aarondeannorman@gmail.com (A.D.N.); 18 Immunology Department, Virgen de las Nieves University Hospital, 18014 Granada, Spain; manevot@ugr.es; 19 Hematology Department, Morales Meseguer University Hospital, 30008 Murcia, Spain; tzuchen82@gmail.com (T.C.-L.); igamez50@gmail.com (I.G.); 20 MSB Medical School Berlin, D-14197 Berlin, Germany; hauke.thomsen@medicalschool-berlin.de; 21 Cancer Prevention and Control Program, Unit of Biomarkers and Susceptibility, Bellvitge Biomedical Research Institute (IDIBELL), Catalan Institute of Oncology, 08907 Barcelona, Spain; 22 Department of Clinical Sciences, Faculty of Medicine, University of Barcelona, 08907 Barcelona, Spain; 23 Epidemiology Unit and Girona Cancer Registry, Oncology Coordination Plan, Department of Health, Autonomous Government of Catalonia, Catalan Institute of Oncology, Girona Biomedical Research Institute (IdiBGi), 17190 Girona, Spain; 24 Department of Nursing, Universitat de Girona, 17007 Girona, Spain; 25 Josep Carreras Leukemia Research Institute, 08916 Girona, Spain; 26 Department of Hematology, University Hospital of Salamanca (HUS/IBSAL), CIBERONC and Cancer Research Institute of Salamanca-IBMCC (USAL-CSIC), 37007 Salamanca, Spain; mgarca1991@gmail.com (M.G.-Á.); migalcoceba@gmail.com (M.A.); 27 Faculty of Medicine, University of Cantabria, 39011 Santander, Spain; 28 Department of Hematology, Experimental Hematology Unit, Vall d’Hebron Institute of Oncology (VHIO), University Hospital Vall d’Hebron, 08035 Barcelona, Spain; anjecayu@gmail.com; 29 Department of Cancer Prevention and Therapy, Medical University of Wrocław, 50-556 Wrocław, Poland; aleksandra.butrym@gmail.com; 30 Division of Computational Genomics, Mayo Clinic, Rochester, MN 85054, USA; slager.susan@mayo.edu; 31 Division of Hematology, Mayo Clinic, Rochester, MN 55905, USA; 32 Division of Cancer Epidemiology, German Cancer Research Center (DKFZ), Im Neuenheimer Feld 280, 69120 Heidelberg, Germany; k.hemminki@dkfz-heidelberg.de; 33 Faculty of Medicine and Biomedical Center in Pilsen, Charles University in Prague, 30605 Pilsen, Czech Republic; 34 Department of Internal Medicine and Radboud Center for Infectious Diseases, Radboud University Medical Center, 6525 GA Nijmegen, The Netherlands; yang.li@helmholtz-hzi.de (Y.L.); mihai.netea@radboudumc.nl (M.G.N.); 35 Centre for Individualised Infection Medicine (CiiM) & TWINCORE, Joint Ventures between the Helmholtz-Centre for Infection Research (HZI) and the Hannover Medical School (MHH), 30625 Hannover, Germany; 36 Division of Cancer Epidemiology and Genetics, National Cancer Institute, National Institutes of Health, Bethesda, MD 20814, USA; berndts@mail.nih.gov; 37 Department for Immunology & Metabolism, Life and Medical Sciences Institute (LIMES), University of Bonn, 53115 Bonn, Germany; 38 Department of Biochemistry and Molecular Biology I, Faculty of Sciences, University of Granada (UGR), 18012 Granada, Spain

**Keywords:** chronic lymphocytic leukemia, overall survival, TTFT, genetic variants, susceptibility, polygenic risk score

## Abstract

Chronic lymphocytic leukemia (CLL) is the most common leukemia among adults worldwide. Although genome-wide association studies (GWAS) have uncovered the germline genetic component underlying CLL susceptibility, the potential use of GWAS-identified risk variants to predict disease progression and patient survival remains unexplored. Here, we evaluated whether 41 GWAS-identified risk variants for CLL could influence overall survival (OS) and disease progression, defined as time to first treatment (TTFT) in a cohort of 1039 CLL cases ascertained through the CRuCIAL consortium. Although this is the largest study assessing the effect of GWAS-identified susceptibility variants for CLL on OS, we only found a weak association of ten single nucleotide polymorphisms (SNPs) with OS (*p* < 0.05) that did not remain significant after correction for multiple testing. In line with these results, polygenic risk scores (PRSs) built with these SNPs in the CRuCIAL cohort showed a modest association with OS and a low capacity to predict patient survival, with an area under the receiver operating characteristic curve (AUROC) of 0.57. Similarly, seven SNPs were associated with TTFT (*p* < 0.05); however, these did not reach the multiple testing significance threshold, and the meta-analysis with previous published data did not confirm any of the associations. As expected, PRSs built with these SNPs showed reduced accuracy in prediction of disease progression (AUROC = 0.62). These results suggest that susceptibility variants for CLL do not impact overall survival and disease progression in CLL patients.

## 1. Introduction

Chronic lymphocytic leukemia (CLL) is the most common form of leukemia among adults worldwide [1], and its global health burden has risen substantially over the past 30 years [2]. CLL remains an incurable disease [3] with a heterogeneous clinical course and a 10-year survival rate ranging from 47.3% to 72.5% in males and 58.2% to 78.7% in females [4]. Traditional clinical prognostic factors include Rai and Binet staging systems, lymphocyte doubling time, cytogenetic alterations, and point mutations, which are used for patient risk stratification and clinical management [5]. Although age, sex, exposure to chemicals, race/ethnicity, and family history of hematological cancers influence the risk of CLL, recent studies have suggested that the combination of these classical factors with genetic markers might help in predicting disease onset and clinical outcome [6]. However, despite the overall success of genome-wide association studies (GWAS) in identifying susceptibility loci for CLL [7,8,9,10], there remains an unmet need to characterize genetic markers associated with disease progression and overall patient survival. Considering this background, we investigated whether GWAS-identified susceptibility variants for CLL could influence the overall survival (OS) of CLL patients and their disease progression, defined as time to first treatment (TTFT). Finally, we explored whether the effect of selected variants on OS and TTFT could be mediated by immune-related processes through a comprehensive battery of functional experiments developed in the 500FG cohort recruited in the context of the Human Functional Project (HFGP).

## 2. Results

CLL patients from the CRuCIAL cohort had a mean age of 65.87 and a male/female ratio of 1.57, which is in line with the worldwide median age of diagnosis and gender distributions (Table 1) [11]. Median follow-up time was 76.77 months without variation of follow-up statistics for censored patients, and the total number of deceased patients was 287. The OS did not differ significantly by country, ruling out the possibility of any deviation due to multicenter randomized patient recruitment. On the other hand, CLL patients with TTFT data in the CRuCIAL cohort had a mean age of 65.03 and a male/female ratio of 1.62. The median time to first treatment was 759.49 days and the number of deceased patients was 111 (Table 1).

Cox regression analyses showed that ten genetic variants within the *CAMK2D*, *CASP8*, *CFLAR*, *CXXC1*, *GPR37*, *IRF8*, *LEF1*, *MYNN*, *PRKD2*, and *TERC* loci were associated with OS at *p* < 0.05 level (Table 2). Although potentially interesting, none of these associations remained significant after correction for multiple testing, which suggested a weak effect (if any) of these genes in determining patient survival. The lack of previous studies assessing the impact of GWAS-identified risk variants on OS hampered the performance of eventual meta-analyses.

As expected, we found a weak association between the weighted and unweighted PRSs and OS of CLL patients (HR = 1.22, *p* = 1.80 × 10^−5^ and HR_Scaled_80%_ = 1.19, *p* = 7.61 × 10^−5^). Weighted and unweighted PRSs increased the capacity to predict OS by only 6–7%, with an area under the receiver operating characteristic curve (AUROC) for the unweighted and weighted PRS of 0.56 and 0.57, respectively (Table 3).

In agreement with these findings, we found that none of these SNPs were correlated with host immune parameters (cQTL data, absolute numbers of 91 blood-derived cell populations, 106 serum immunological proteins, or 7 steroid hormones), which reinforced the hypothesis of a null effect of these markers in determining overall patient survival.

On the other hand, Cox regression analyses adjusted for age, sex, and country of origin revealed that seven genetic variants within the *ACOXL*, *CASP8*, *GRAMD1B*, *MYNN*, *PRKD2*, *TERC*, and *ZBTB7A|MAP2K2* loci were associated with TTFT at *p* < 0.05 level (Table 4). However, none of the associations with TTFT remained significant after correction for multiple testing, suggesting that these susceptibility variants for CLL do not have a relevant role in determining disease progression.

In line with these data, a meta-analysis of our data including data from a previous GWAS confirmed that none of these loci have a significant impact on TTFT (Table 5). These findings support the notion of a null effect of susceptibility variants on disease progression in CLL.

As expected, the association of weighted and unweighted PRSs built with those variants associated with TTFT in the CRuCIAL cohort was very modest (HR_Unweighted_ = 1.26, *p* = 6.20 × 10^−4^ and HR_Weighted_ = 1.32, *p* = 5.17 × 10^−4^ and HR_Unweighted_Scaled_80% =_ 1.29, *p* = 4.40 × 10^−5^ and HR_Weighted_Scaled_80% =_ 1.34, *p* = 6.60 × 10^−5^). Therefore, we were able to confirm that these PRSs were not useful in accurately predicting disease progression (AUROC_Unweighted_ = 0.59, AUROC_Weighted_ = 0.60, AUROC_Unweighted_Scaled_80%_ = 0.61 and AUROC_Weighted_Scaled_80%_ = 0.61; Table 6).

## 3. Discussion

This is the largest study evaluating the association of GWAS-identified susceptibility variants for CLL with OS, and one of the first studies assessing the effect of GWAS-identified susceptibility variants for CLL in disease progression. Even though we found potentially interesting associations between ten SNPs within the *CAMK2D*, *CASP8*, *CFLAR*, *CXXC1*, *GPR37*, *IRF8*, *LEF1*, *MYNN*, *PRKD2*, and *TERC* loci and the OS of CLL patients, none of these associations remained significant after correction for multiple testing. As expected, we found a modest association between weighted and unweighted PRSs and OS, which increased the prediction capacity by only 7%. These findings suggest that susceptibility variants for CLL do not have a large influence on OS, which is in agreement with a previous study that, using a similar approach, demonstrated that susceptibility variants do not influence the OS of patients diagnosed with multiple myeloma, another B cell malignancy [13].

This study failed to find a statistically significant association between GWAS-identified risk variants for CLL and TTFT. We found that only seven SNPs within the *ACOXL*, *CASP8*, *GRAMD1B*, *MYNN*, *PRKD2*, *TERC*, and *ZBTB7A|MAP2K2* loci showed an association with TTFT at *p* < 0.05 level. None of these associations remained significant after correction for multiple testing, and a meta-analysis of our data with those from a previous GWAS on TTFT confirmed the null effect of these variants on disease progression. In agreement with these results, we found that weighted and unweighted PRSs did not have the capacity to predict TTFT. Nonetheless, in light of the relatively low power of our study (80% of power to detect an HR of 1.45 for a SNP with a frequency of 0.25) and the sparse number of studies assessing the impact of GWAS-identified risk variants for CLL on OS and TTFT, we cannot rule out the possibility that some of these SNPs might have a stronger effect on the modulation of OS and disease progression. In fact, we found it interesting that carriers of the *CXXC1*_rs1036935A_ allele had poorer OS, as our team has previously reported that the presence of this allele is associated with decreased numbers of CD19+CD20+ B cells [14], a subtype of cells poorly expressed in CLL patients. The *CXXC1* locus encodes for a protein of the SETD1 complex, which acts as an epigenetic transcriptional activator; if deregulated, it can lead to tumor progression and poorer survival [15].

This study has both strengths and weaknesses. The major strengths of this study are the large number of genetic markers analyzed and the relatively large size of the study population. Another strength is the comprehensive functional analysis conducted in the HFGP cohort, which included cQTL data after stimulation of whole blood, PBMCs, and MDMs with LPS, PHA, Pam3Cys, CpG, and *B. burgdorferi* and *E. coli*, as well as data on serological and plasmatic inflammatory proteins, serum steroid hormones, and blood-derived immune cell types. A limitation of this study is its multicentric nature, with inevitable drawbacks such as the impossibility of uniformly collecting medication history and Rai–Binet status for a significant proportion of the patients analyzed. In addition, considering that all study participants included in this study were of European ancestry, we could not determine the impact of GWAS-identified variants on patient survival and TTFT in other ethnic or ancestral populations.

## 4. Materials and Methods

### 4.1. Study Participants

This study included 1039 CLL patients ascertained through the CRuCIAL consortium. CLL patients were diagnosed following the updated international criteria [5]. Study participants were of European ancestry, and provided their written informed consent to participate in the study, which was approved by the ethical review committee of participant institutions: Virgen de las Nieves University Hospital (Granada, Spain, 0760-N-18); University Hospital of Salamanca (Salamanca, Spain, PI90/07/2018); Hospital del Mar (Barcelona, Spain); Catalan Institute of Oncology (Barcelona, Spain); Morales Meseguer University Hospital (Murcia, Spain); Consortium for Biomedical Research in Epidemiology and Public Health (CIBERESP) group (Spain); University of Modena and Reggio Emilia, AOU Policlinico (Modena, Italy); University of Pisa (Pisa, Italy), Wroclaw Medical University (Wroclaw, Poland); and the Radboud University Medical Center (Nijmegen, The Netherlands, 2011/299). A detailed description of the study cohort has been reported elsewhere [14]. The main characteristics of the patients are included in Table 1. This study followed the Declaration of Helsinki.

### 4.2. SNP Selection and Genotyping

A total of 41 single nucleotide polymorphisms (SNPs) were selected based on published GWAS, functionality data, and linkage disequilibrium between the SNPs (Supplementary Table S1) [14]. Genotyping of selected SNPs was carried out at GENYO (Centre for Genomics and Oncological Research: Pfizer/University of Granada/Andalusian Regional Government, Granada, Spain) using KASPar^®^ assays (LGC Genomics, Hoddesdon, UK) according to previously reported protocols. For internal quality control, ~5% of samples were randomly selected and included as duplicates. Concordance between the original and the duplicate samples for the selected SNPs was ≥99.0%. Call rate was higher than 90%.

### 4.3. Statistical Analysis and Meta-Analysis

The Hardy–Weinberg Equilibrium (HWE) test was performed in the control group using a standard observed–expected chi-square (χ^2^) test. The primary outcome was OS and the endpoint was defined as death from any cause. Survival time was calculated as the time from CLL diagnosis until the occurrence of the study endpoint, censoring at the date of death or the last observed follow-up time. The second outcome was time to first treatment (TTFT), defined as the interval between CLL diagnosis and date of the first treatment or last follow-up, while the endpoint was defined as death from any cause. Association with OS and TTFT, defined as hazard ratio (HR), was calculated for each SNP using Cox regression analysis adjusted for age, sex, and country of origin. Considering the number of SNPs and the number of inheritance models tested (log-additive, dominant, and recessive), we set a significance threshold to 0.00041 (0.05/41/3) using the Bonferroni correction. Association analyses were performed using STATA (v12.1; Stata Corp, College Station, TX, USA) and power calculations were estimated using the survSNP package in R (v4.1.1; R Core Team, 2018).

In order to confirm potentially interesting associations with disease progression, we conducted a meta-analysis of the CRuCIAL data with those from a previous GWAS [12] using METAL and assuming a fixed-effect model; the I^2^ statistic was used to assess statistical heterogeneity between cohorts. Next, in order to confirm whether susceptibility variants could predict OS and disease progression, we computed weighted and unweighted polygenic risk scores (PRSs) considering those SNPs associated with OS and TTFT at a threshold of *p* < 0.05. We built PRSs considering either subjects with a call rate of 100% (n = 891 and 290) or 80% (n = 1003 and 323) for OS and TTFT, respectively. PRS is an approach that estimates the individual risk to a phenotype or disease, which is calculated as a sum of their genotypes weighted by corresponding genotype effect sizes from summary statistic data. A detailed explanation of how the PRS scores were generated has been published in [16].

### 4.4. Functional Effect of the GWAS-Identified Risk Variants on Immune Responses

Mechanistically, we investigated whether selected SNPs were correlated with production of nine cytokines after in vitro stimulation of peripheral mononuclear cells (PBMCs) from 408 healthy subjects from the Human Functional Genomic Project (HFGP) cohort with LPS (100 ng/mL, Sigma-Aldrich, St. Louis, MO, USA), PHA (10 μg/mL, Sigma-Aldrich, St. Louis, MO, USA), Pam3Cys (10 μg/mL, EMC microcollections, Tuebingen, Germany), CpG (100 ng/mL, InvivoGen, San Diego, CA, USA), and *B. burgdorferi* (ATCC strain 35210) and *E. coli* (ATCC 25922). In addition, we investigated the correlation between SNPs and circulating concentrations of 103 serum and plasmatic inflammatory proteins, absolute numbers of 91 blood-derived immune cell populations (Supplementary Tables S2 and S3) and 7 plasma steroid hormones. Experimental protocols for the functional experiments have been previously described in detail [17,18]. Functional results for selected SNPs have been previously published by our team as part of a study in the context of the CRuCIAL consortium that aimed at validating the associations of GWAS-identified risk variants for CLL [14].

## 5. Conclusions

This study suggests that susceptibility variants for CLL do not substantially impact the overall survival of CLL patients, and confirms previous results suggesting the null effect of these variants on TTFT.

## Figures and Tables

**Table 1:** Patient characteristics of the CRuCIAL cohort used for OS and TTFT analyses.

CRuCIAL Cohort (1039 CLL Cases for OS Analysis)		CRuCIAL Cohort (354 CLL Cases for TTFT Analysis)	
Age (years)	65.87 ± 11.05	Age (years)	65.03 ± 10.94
Sex ratio (male/female)	1.57 (636/403)	Sex ratio (male/female)	1.62 (219/135)
Country of origin (alive/decesead)		Country of origin	
Spain	754 (536/218)	Spain	227 (174/73)
Italy	285 (216/69)	Italy	127 (86/41)
Median follow-up time (months)	76.77 (50–123)	Median TTFT (days)	759.49 (31–1148.74)
Alive	76 (52.1–123)	Alive	830.81 (44.13–1308.93)
Deceased	77 (44–123)	Deceased	564.83 (13.10–800)
Status at follow-up		Status at follow-up	
Alive	752 (72.38)	Alive	239 (68.29)
Deceased	287 (27.62)	Deceased	111 (31.71)
Binet Stage		Binet stage	
A	647 (81.48)	A	201 (64.84)
B	99 (12.46)	B	68 (21.94)
C	48 (06.04)	C	41 (13.23)
Rai Stage		Rai stage	
0	597 (65.67)	0	124 (37.35)
I	152 (16.72)	I	105 (31.63)
II	114 (12.57)	II	63 (18.98)
III	12 (01.31)	III	16 (04.82)
IV	34 (03.73)	IV	24 (07.23)

Data are mean ± standard deviation, n (%), or percentiles (25th–75th percentiles).

**Table 2:** Association of GWAS-identified risk variants for CLL and OS.

SNP	Chr.	Nearby Gene	Risk Allele	HR (95%CI) ^δ^	*p*	HR (95%CI) ^Ϯ^	*p*	HR (95%CI) ^¥^	*p*
rs4368253	18	*AC107990.1||NFE2L3P1*	C	0.94 (0.78–1.14)	0.539	0.93 (0.59–1.45)	0.746	0.93 (0.73–1.18)	0.545
rs1439287	2	*ACOXL*	T	1.03 (0.88–1.21)	0.700	0.99 (0.76–1.29)	0.957	1.10 (0.84–1.44)	0.481
rs58055674	2	*ACOXL*	C	1.08 (0.90–1.30)	0.424	1.12 (0.89–1.42)	0.335	1.02 (0.64–1.61)	0.936
rs7944004	11	*ASCL2||C11orf21*	T	0.97 (0.82–1.15)	0.735	1.06 (0.79–1.43)	0.699	0.89 (0.69–1.16)	0.394
rs4987855	18	*BCL2*	G	0.97 (0.71–1.32)	0.848	0.68 (0.10–4.87)	0.701	0.98 (0.71–1.34)	0.886
rs2651823	11	*C11orf21|TSPAN32*	A	0.95 (0.81–1.12)	0.523	1.03 (0.78–1.36)	0.857	0.85 (0.65–1.11)	0.233
rs1476569	4	*CAMK2D*	G	1.12 (0.95–1.32)	0.176	1.31 (1.03–1.67)	0.028	0.92 (0.64–1.32)	0.643
rs3769825	2	*CASP8*	T	1.20 (1.02–1.43)	0.033	1.23 (0.93–1.63)	0.150	1.32 (1.01–1.73)	0.041
rs7558911	2	*CFLAR*	A	1.19 (1.01–1.41)	0.040	1.22 (0.91–1.63)	0.175	1.30 (1.00–1.67)	0.046
rs1036935	18	*CXXC1*	A	1.27 (1.07–1.51)	0.008	1.34 (1.06–1.70)	0.015	1.43 (0.98–2.10)	0.066
rs1359742	9	*DMRTA1*	G	1.05 (0.89–1.23)	0.575	1.02 (0.78–1.34)	0.875	1.11 (0.85–1.45)	0.441
rs6546149	2	*DTNB*	G	1.09 (0.91–1.31)	0.356	1.06 (0.84–1.35)	0.607	1.29 (0.86–1.93)	0.222
rs9880772	3	*EOMES|LINC01980*	T	0.98 (0.84–1.14)	0.787	1.04 (0.80–1.34)	0.787	0.90 (0.69–1.18)	0.455
rs13015798	2	*FAM126B*	A	1.06 (0.88–1.28)	0.548	1.00 (0.64–1.54)	0.989	1.10 (0.87–1.39)	0.444
rs6586163	10	*FAS*	A	0.99 (0.84–1.17)	0.916	1.08 (0.80–1.45)	0.631	0.92 (0.71–1.20)	0.554
rs2267708	7	*GPR37*	T	0.86 (0.72–1.02)	0.081	0.91 (0.70–1.19)	0.504	0.70 (0.51–0.97)	0.030
rs2953196	11	*GRAMD1B*	G	0.85 (0.70–1.04)	0.123	0.70 (0.42–1.17)	0.176	0.85 (0.67–1.09)	0.203
rs35923643	11	*GRAMD1B*	G	0.88 (0.72–1.07)	0.192	0.80 (0.63–1.02)	0.068	1.12 (0.71–1.78)	0.631
rs3800461	6	*ILRUN*	C	0.92 (0.67–1.27)	0.620	0.91 (0.65–1.27)	0.562	1.20 (0.30–4.86)	0.795
rs9392504	6	*IRF4*	A	0.88 (0.74–1.04)	0.143	0.80 (0.60–1.08)	0.145	0.88 (0.68–1.13)	0.322
rs391855	16	*IRF8*	A	0.87 (0.74–1.03)	0.104	0.99 (0.72–1.36)	0.946	0.74 (0.58–0.96)	0.021
rs898518	4	*LEF1*	A	1.12 (0.94–1.33)	0.215	1.50 (1.00–2.26)	0.049	1.04 (0.82–1.33)	0.721
rs34676223	1	*MDS2*	C	0.99 (0.83–1.18)	0.899	0.88 (0.61–1.27)	0.491	1.03 (0.81–1.30)	0.803
rs57214277	4	*MYL12BP2||LINC02363*	T	1.02 (0.86–1.21)	0.805	1.01 (0.79–1.29)	0.926	1.06 (0.77–1.46)	0.729
rs10936599	3	*MYNN*	C	0.91 (0.74–1.11)	0.344	0.57 (0.36–0.90)	0.016	0.99 (0.77–1.26)	0.922
rs11715604	3	*NCK1*	T	0.98 (0.80–1.20)	0.831	1.06 (0.61–1.85)	0.842	0.96 (0.74–1.23)	0.723
rs6489882	12	*OAS3*	G	1.09 (0.92–1.29)	0.309	1.16 (0.90–1.50)	0.240	1.06 (0.78–1.45)	0.696
rs140522	22	*ODF3B*	T	1.02 (0.86–1.20)	0.854	1.11 (0.87–1.41)	0.410	0.87 (0.61–1.24)	0.431
rs2236256	6	*OPRM1||IPCEF1*	C	1.03 (0.87–1.21)	0.752	0.96 (0.74–1.23)	0.729	1.14 (0.86–1.51)	0.348
rs11637565	15	*PCAT29|LOC107984788*	G	0.97 (0.82–1.16)	0.753	0.96 (0.74–1.25)	0.753	0.97 (0.71–1.33)	0.842
rs17246404	7	*POT1*	C	0.95 (0.79–1.14)	0.572	0.95 (0.61–1.47)	0.818	0.93 (0.74–1.18)	0.557
rs2511714	8	*POU5F1P2||ODF1*	G	0.91 (0.77–1.08)	0.268	0.92 (0.72–1.17)	0.485	0.82 (0.59–1.14)	0.240
rs11083846	19	*PRKD2*	A	1.20 (0.99–1.45)	0.061	1.16 (0.92–1.47)	0.209	1.62 (1.06–2.50)	0.027
rs888096	2	*QPCT||RNU6-1116P*	A	0.95 (0.81–1.13)	0.590	0.97 (0.76–1.24)	0.837	0.88 (0.63–1.23)	0.454
rs41271473	1	*RHOU*	G	0.96 (0.74–1.25)	0.770	1.46 (0.64–3.32)	0.366	0.88 (0.64–1.21)	0.432
rs73718779	6	*SERPINB6*	A	1.03 (0.78–1.37)	0.823	1.04 (0.77–1.40)	0.806	0.97 (0.24–3.94)	0.970
rs12638862	3	*TERC*	A	0.89 (0.73–1.08)	0.229	0.62 (0.40–0.97)	0.037	0.94 (0.74–1.19)	0.602
rs7705526	5	*TERT*	A	1.07 (0.90–1.27)	0.468	1.00 (0.78–1.28)	0.997	1.25 (0.91–1.70)	0.163
rs61904987	11	*TMPRSS5||DRD2*	T	0.94 (0.71–1.23)	0.632	0.94 (0.70–1.28)	0.712	0.73 (0.23–2.30)	0.588
rs926070	6	*TSBP1-AS1*	A	1.11 (0.92–1.33)	0.285	1.19 (0.77–1.82)	0.433	1.12 (0.88–1.42)	0.343
rs7254272	19	*ZBTB7A|MAP2K2*	A	0.82 (0.66–1.02)	0.078	0.78 (0.61–1.00)	0.051	0.92 (0.50–1.69)	0.794

Abbreviations: SNP, single nucleotide polymorphism; HR, hazards ratio. Significant results in bold (*p* < 0.05). ^δ^ Cox regression analysis assuming a log-additive model of inheritance. ^Ϯ^ Cox regression analysis assuming a dominant model. ^¥^ Cox regression analysis assuming a recessive model.

**Table 3:** Associations between unweighted and weighted PRSs and OS.

Polygenic Risk Scores (n = 891)				AUROC
	Quintiles	HR 95%CI ^a^	*p*	AUROC (95%CI)
Unweighted, subjects with 100% call rate	1	1.00	-	
	2	1.06 (0.70–1.60)	0.787	
	3	1.67 (1.15–2.43)	0.007	
	4	1.42 (0.99–2.03)	0.053	
	5	2.36 (1.56–3.58)	5.30 × 10^−5^	
	Continuous ^b^	1.20 (1.09–1.31)	8.70 × 10^−5^	0.56 (0.52–0.60)
Weighted, subjects with 100% call rate	1	1.00	-	
	2	1.33 (0.85–2.09)	0.206	
	3	2.05 (1.34–3.15)	0.001	
	4	1.68 (1.08–2.59)	0.020	
	5	2.50 (1.63–3.83)	2.40 × 10^−5^	
	Continuous ^b^	1.22 (1.11–1.33)	1.80 × 10^−5^	0.57 (0.53–0.61)
Polygenic Risk Scores (n = 1003)				AUROC
	Quintiles	HR 95%CI ^a^	*p*	AUROC (95%CI)
Unweighted, subjects with 80% call rate	1	1.00	-	
	2	0.99 (0.66–1.46)	0.948	
	3	1.48 (1.05–2.11)	0.027	
	4	1.36 (0.98–1.90)	0.066	
	5	2.08 (1.41–3.07)	2.41 × 10^−4^	
	Continuous ^b^	1.17 (1.08–1.28)	2.32 × 10^−4^	0.55 (0.51–0.59)
Weighted, subjects with 80% call rate	1	1.00	-	
	2	1.29 (0.85–1.95)	0.224	
	3	1.78 (1.19–2.67)	0.005	
	4	1.57 (1.05–2.35)	0.028	
	5	2.19 (1.48–3.26)	9.80 × 10^−5^	
	Continuous ^b^	1.19 (1.09–1.29)	7.61 × 10^−5^	0.56 (0.52–0.60)

^a^ HR, hazards ratio; CI, confidence interval. All analyses were adjusted for age, sex, and country of origin; ^b^ The unit for the analysis with the continuous variable was the increment of one quintile.

**Table 4:** Association of GWAS-identified risk variants for CLL and TTFT.

SNP	Chr.	Nearby Gene	Risk Allele	HR (95%CI) ^δ^	*p*	HR (95%CI) ^Ϯ^	*p*	HR (95%CI) ^¥^	*p*
rs4368253	18	*AC107990.1||NFE2L3P1*	C	1.03 (0.74–1.42)	0.871	0.62 (0.31–1.25)	0.184	1.18 (0.80–1.74)	0.413
rs1439287	2	*ACOXL*	T	1.05 (0.81–1.37)	0.708	0.98 (0.65–1.50)	0.943	1.18 (0.76–1.83)	0.469
rs58055674	2	*ACOXL*	C	1.39 (1.02–1.90)	0.036	1.60 (1.08–2.38)	0.019	1.18 (0.51–2.73)	0.696
rs7944004	11	*ASCL2||C11orf21*	T	1.04 (0.78–1.39)	0.769	1.01 (0.61–1.67)	0.967	1.09 (0.71–1.68)	0.684
rs4987855	18	*BCL2*	G	0.75 (0.44–1.27)	0.281	NA	NA	0.73 (0.43–1.25)	0.256
rs2651823	11	*C11orf21|TSPAN32*	A	1.12 (0.85–1.46)	0.418	1.13 (0.73–1.76)	0.591	1.20 (0.77–1.85)	0.423
rs1476569	4	*CAMK2D*	G	1.01 (0.76–1.35)	0.951	1.02 (0.69–1.51)	0.915	0.99 (0.54–1.82)	0.971
rs3769825	2	*CASP8*	T	1.41 (1.06–1.87)	0.017	1.56 (0.98–2.47)	0.059	1.58 (1.00–2.48)	0.048
rs7558911	2	*CFLAR*	A	1.21 (0.93–1.58)	0.163	1.23 (0.78–1.95)	0.375	1.35 (0.89–2.05)	0.155
rs1036935	18	*CXXC1*	A	1.13 (0.83–1.53)	0.446	1.14 (0.77–1.69)	0.503	1.24 (0.59–2.61)	0.568
rs1359742	9	*DMRTA1*	G	0.98 (0.74–1.30)	0.884	0.85 (0.54–1.32)	0.468	1.11 (0.72–1.73)	0.636
rs6546149	2	*DTNB*	G	0.95 (0.69–1.31)	0.769	0.90 (0.61–1.33)	0.587	1.14 (0.55–2.36)	0.723
rs9880772	3	*EOMES|LINC01980*	T	0.96 (0.73–1.24)	0.735	0.96 (0.64–1.43)	0.831	0.91 (0.56–1.48)	0.717
rs13015798	2	*FAM126B*	A	0.92 (0.68–1.23)	0.567	0.88 (0.45–1.70)	0.695	0.90 (0.61–1.33)	0.605
rs6586163	10	*FAS*	A	0.81 (0.62–1.07)	0.139	0.80 (0.51–1.26)	0.339	0.72 (0.45–1.13)	0.150
rs2267708	7	*GPR37*	T	0.76 (0.58–1.00)	0.052	0.69 (0.46–1.03)	0.069	0.71 (0.43–1.16)	0.172
rs2953196	11	*GRAMD1B*	G	0.82 (0.59–1.14)	0.240	0.85 (0.34–2.10)	0.724	0.76 (0.50–1.16)	0.202
rs35923643	11	*GRAMD1B*	G	0.71 (0.52–0.98)	0.040	0.68 (0.46–1.02)	0.061	0.54 (0.23–1.28)	0.164
rs3800461	6	*ILRUN*	C	0.97 (0.61–1.54)	0.881	0.94 (0.55–1.60)	0.808	1.17 (0.27–5.08)	0.832
rs9392504	6	*IRF4*	A	0.94 (0.72–1.23)	0.648	1.07 (0.63–1.81)	0.800	0.83 (0.55–1.26)	0.380
rs391855	16	*IRF8*	A	1.14 (0.87–1.49)	0.336	1.28 (0.77–2.14)	0.341	1.15 (0.77–1.72)	0.503
rs898518	4	*LEF1*	A	1.16 (0.87–1.54)	0.310	1.58 (0.82–3.04)	0.172	1.09 (0.74–1.62)	0.650
rs34676223	1	*MDS2*	C	1.28 (0.96–1.71)	0.098	1.96 (0.94–4.07)	0.073	1.24 (0.84–1.82)	0.280
rs57214277	4	*MYL12BP2||LINC02363*	T	1.10 (0.85–1.43)	0.456	1.28 (0.86–1.91)	0.225	0.97 (0.58–1.61)	0.897
rs10936599	3	*MYNN*	C	1.03 (0.73–1.46)	0.866	0.42 (0.21–0.83)	0.013	1.30 (0.85–1.98)	0.222
rs11715604	3	*NCK1*	T	0.78 (0.55–1.10)	0.158	0.59 (0.24–1.48)	0.263	0.77 (0.51–1.17)	0.225
rs6489882	12	*OAS3*	G	1.01 (0.76–1.34)	0.929	1.06 (0.70–1.59)	0.797	0.96 (0.56–1.63)	0.868
rs140522	22	*ODF3B*	T	0.88 (0.66–1.18)	0.391	0.86 (0.58–1.28)	0.452	0.81 (0.43–1.53)	0.523
rs2236256	6	*OPRM1||IPCEF1*	C	1.06 (0.79–1.41)	0.704	0.79 (0.52–1.21)	0.279	1.50 (0.97–2.33)	0.070
rs11637565	15	*PCAT29|LOC107984788*	G	0.90 (0.68–1.20)	0.482	1.04 (0.67–1.61)	0.874	0.67 (0.38–1.16)	0.155
rs17246404	7	*POT1*	C	1.14 (0.83–1.56)	0.425	1.63 (0.70–3.79)	0.257	1.08 (0.72–1.61)	0.702
rs2511714	8	*POU5F1P2||ODF1*	G	0.97 (0.72–1.31)	0.838	0.93 (0.61–1.40)	0.721	1.03 (0.57–1.86)	0.914
rs11083846	19	*PRKD2*	A	1.34 (1.00–1.80)	0.050	1.21 (0.82–1.78)	0.331	2.31 (1.31–4.08)	0.004
rs888096	2	*QPCT||RNU6-1116P*	A	1.02 (0.76–1.35)	0.912	0.96 (0.64–1.44)	0.851	1.14 (0.66–1.96)	0.631
rs41271473	1	*RHOU*	G	0.76 (0.50–1.13)	0.176	0.85 (0.26–2.76)	0.786	0.69 (0.42–1.13)	0.139
rs73718779	6	*SERPINB6*	A	1.15 (0.74–1.78)	0.536	1.09 (0.68–1.74)	0.734	2.86 (0.69–11.9)	0.148
rs12638862	3	*TERC*	A	0.94 (0.66–1.33)	0.715	0.40 (0.19–0.84)	0.015	1.12 (0.74–1.69)	0.601
rs7705526	5	*TERT*	A	0.92 (0.70–1.23)	0.589	0.79 (0.53–1.17)	0.240	1.13 (0.69–1.86)	0.633
rs61904987	11	*TMPRSS5||DRD2*	T	1.28 (0.86–1.92)	0.228	1.35 (0.85–2.16)	0.203	1.23 (0.30–5.10)	0.776
rs926070	6	*TSBP1-AS1*	A	1.00 (0.74–1.35)	0.987	0.87 (0.45–1.69)	0.684	1.04 (0.71–1.54)	0.835
rs7254272	19	*ZBTB7A|MAP2K2*	A	0.74 (0.51–1.07)	0.110	0.62 (0.41–0.96)	0.030	1.34 (0.58–3.09)	0.497

Abbreviations: SNP, single nucleotide polymorphism; HR, hazards ratio. Significant results in bold (*p* < 0.05). ^δ^ Cox regression analysis assuming a log-additive model of inheritance. ^Ϯ^ Cox regression analysis assuming a dominant model of inheritance. ^¥^ Cox regression analysis assuming a recessive model of inheritance.

**Table 5:** Meta-analysis of association estimates of GWAS-identified risk variants for CLL and disease progression in the CRuCIAL cohorts with a previous GWAS.

SNP	Chr.	Nearby Gene	Risk Allele	CRuCIAL Consortium (354 CLL Cases)		Lin et al. (2021) [12] (755 CLL Cases)		Meta-Analysis (1109 CLL Cases)		
				HR (95%CI) ^δ^	*p*	HR (95%CI) ^δ^	*p*	HR (95%CI) ^δ^	*p*	*p_het_*
rs4368253	18	*AC107990.1||NFE2L3P1*	C	1.03 (0.74–1.42)	0.871	1.03 (0.87–1.17)	0.684	1.03 (0.85–1.19)	0.726	0.985
rs1439287	2	*ACOXL*	T	1.05 (0.81–1.37)	0.708	1.00 (0.86–1.12)	0.990	1.01 (1.16–0.88)	0.858	0.765
rs58055674	2	*ACOXL*	C	1.39 (1.02–1.90)	0.036	0.98 (0.84–1.15)	0.837	1.08 (0.89–1.23)	0.398	0.096
rs7944004	11	*ASCL2||C11orf21*	T	1.04 (0.78–1.39)	0.769	-	-	1.04 (1.35–0.80)	0.783	1.000
rs4987855	18	*BCL2*	G	0.75 (0.44–1.27)	0.281	0.82 (0.45–1.10)	0.230	0.78 (0.33–1.10)	0.205	0.734
rs2651823	11	*C11orf21|TSPAN32*	A	1.12 (0.85–1.46)	0.418	0.93 (0.80–1.07)	0.302	0.98 (1.14–0.84)	0.796	0.303
rs1476569	4	*CAMK2D*	G	1.01 (0.76–1.35)	0.951	0.99 (0.85–1.15)	0.898	1.00 (0.83–1.14)	0.955	0.917
rs3769825	2	*CASP8*	T	1.41 (1.06–1.87)	0.017	1.10 (0.96–1.22)	0.149	1.01 (1.18–0.87)	0.870	0.018
rs7558911	2	*CFLAR*	A	1.21 (0.93–1.58)	0.163	0.81 (0.63–0.97)	0.019	0.94 (1.10–0.80)	0.421	0.048
rs1036935	18	*CXXC1*	A	1.13 (0.83–1.53)	0.446	0.96 (0.83–1.13)	0.660	1.01 (1.19–0.85)	0.949	0.444
rs1359742	9	*DMRTA1*	G	0.98 (0.74–1.30)	0.884	0.93 (0.76–1.07)	0.344	0.94 (0.77–1.10)	0.506	0.778
rs6546149	2	*DTNB*	G	0.95 (0.69–1.31)	0.769	0.86 (0.73–1.01)	0.067	0.87 (0.64–1.06)	0.192	0.652
rs9880772	3	*EOMES|LINC01980*	T	0.96 (0.73–1.24)	0.735	1.00 (0.87–1.12)	0.954	0.98 (1.13–0.86)	0.828	0.815
rs13015798	2	*FAM126B*	A	0.92 (0.68–1.23)	0.567	0.78 (0.57–0.96)	0.015	0.85 (1.02–0.71)	0.076	0.556
rs6586163	10	*FAS*	A	0.81 (0.62–1.07)	0.139	1.00 (0.86–1.13)	0.972	0.95 (1.11–0.82)	0.527	0.249
rs2267708	7	*GPR37*	T	0.76 (0.58–1.00)	0.052	0.98 (0.85–1.12)	0.743	0.91 (1.07–0.78)	0.273	0.176
rs2953196	11	*GRAMD1B*	G	0.82 (0.59–1.14)	0.240	1.16 (0.99–1.31)	0.065	1.06 (0.84–1.23)	0.596	0.102
rs35923643	11	*GRAMD1B*	G	0.71 (0.52–0.98)	0.040	1.18 (1.00–1.39)	0.049	1.03 (0.84–1.19)	0.760	0.021
rs3800461	6	*ILRUN*	C	0.97 (0.61–1.54)	0.881	1.19 (0.96–1.47)	0.105	1.12 (1.41–0.88)	0.363	0.426
rs9392504	6	*IRF4*	A	0.94 (0.72–1.23)	0.648	1.03 (0.88–1.16)	0.678	1.00 (1.16–0.87)	0.970	0.597
rs391855	16	*IRF8*	A	1.14 (0.87–1.49)	0.336	1.00 (0.85–1.13)	0.967	1.04 (1.21–0.89)	0.624	0.477
rs898518	4	*LEF1*	A	1.16 (0.87–1.54)	0.310	0.92 (0.76–1.06)	0.282	0.98 (1.14–0.84)	0.818	0.234
rs34676223	1	*MDS2*	C	1.28 (0.96–1.71)	0.098	1.06 (0.90–1.19)	0.467	1.11 (0.93–1.25)	0.210	0.347
rs57214277	4	*MYL12BP2||LINC02363*	T	1.10 (0.85–1.43)	0.456	0.92 (0.79–1.06)	0.228	0.97 (1.13–0.84)	0.728	0.286
rs10936599	3	*MYNN*	C	1.03 (0.73–1.46)	0.866	0.91 (0.70–1.08)	0.314	0.94 (0.72–1.13)	0.586	0.587
rs11715604	3	*NCK1*	T	0.78 (0.55–1.10)	0.158	1.05 (0.87–1.27) ^η^	0.614	0.96 (0.73–1.15)	0.719	0.207
rs6489882	12	*OAS3*	G	1.01 (0.76–1.34)	0.929	1.10 (0.95–1.27)	0.213	1.06 (0.91–1.18)	0.450	0.588
rs140522	22	*ODF3B*	T	0.88 (0.66–1.18)	0.391	1.04 (0.90–1.20)	0.641	0.99 (1.16–0.85)	0.927	0.397
rs2236256	6	*OPRM1||IPCEF1*	C	1.06 (0.79–1.41)	0.704	1.04 (0.91–1.20)	0.568	1.04 (0.88–1.18)	0.580	0.930
rs11637565	15	*PCAT29|LOC107984788*	G	0.90 (0.68–1.20)	0.482	0.89 (0.77–1.03)	0.112	0.88 (0.68–1.05)	0.182	0.958
rs17246404	7	*POT1*	C	1.14 (0.83–1.56)	0.425	1.01 (0.85–1.15)	0.872	1.04 (0.86–1.19)	0.636	0.577
rs2511714	8	*POU5F1P2||ODF1*	G	0.97 (0.72–1.31)	0.838	1.00 (0.87–1.14)	0.962	0.99 (0.83–1.13)	0.895	0.883
rs11083846	19	*PRKD2*	A	1.34 (1.00–1.80)	0.050	1.11 (0.94–1.30)	0.228	1.17 (1.41–0.98)	0.088	0.342
rs888096	2	*QPCT||RNU6-1116P*	A	1.02 (0.76–1.35)	0.912	1.08 (0.93–1.24)	0.324	1.06 (1.26–0.90)	0.484	0.801
rs41271473	1	*RHOU*	G	0.76 (0.50–1.13)	0.176	0.92 (0.71–1.09)	0.368	0.87 (0.61–1.08)	0.247	0.453
rs73718779	6	*SERPINB6*	A	1.15 (0.74–1.78)	0.536	0.74 (0.42–0.99)	0.040	1.23 (1.59–0.95)	0.113	0.750
rs12638862	3	*TERC*	A	0.94 (0.66–1.33)	0.715	0.95 (0.76–1.11)	0.567	0.95 (1.14–0.79)	0.582	0.958
rs7705526	5	*TERT*	A	0.92 (0.70–1.23)	0.589	-	-	0.92 (1.27–0.67)	0.621	1.000
rs61904987	11	*TMPRSS5||DRD2*	T	1.28 (0.86–1.92)	0.228	1.06 (0.87–1.29)	0.570	1.11 (1.40–0.89)	0.357	0.481
rs926070	6	*TSBP1-AS1*	A	1.00 (0.74–1.35)	0.987	1.07 (0.91–1.19)	0.382	1.05 (1.25–0.89)	0.570	0.742
rs7254272	19	*ZBTB7A|MAP2K2*	A	0.74 (0.51–1.07)	0.110	1.12 (0.93–1.35)	0.242	1.00 (1.22–0.82)	0.991	0.096

Abbreviations: SNP, single nucleotide polymorphism; HR, hazards ratio. CI, confidence interval; Meta-analysis was performed assuming a fixed-effect model. Significant results in bold (*p* < 0.05). ^η^ Authors report the effect found for the rs62410363 (a SNP in strong linkage disequilibrium with the rs11715604, r2 = 0.97). ^δ^ Cox regression analyses were adjusted for age, sex, and country of origin and were calculated according to log-additive model of inheritance.

**Table 6:** Associations between unweighted and weighted PRSs and disease progression.

Polygenic Risk Scores (n = 290)				AUROC
	Quintiles	HR 95%CI ^a^	*p*	AUROC (95%CI)
Unweighted, subjects with 100% call rate	1	1.00	-	
	2	1.23 (0.68–2.22)	0.487	
	3	-	-	
	4	1.89 (1.08–3.31)	0.026	
	5	2.66 (1.45–4.88)	1.50 × 10^−3^	
	Continuous ^b^	1.26 (1.11–1.45)	6.20 × 10^−4^	0.59 (0.52–0.66)
Weighted, subjects with 100% call rate	1	1.00	-	
	2	2.22 (1.05–4.71)	0.037	
	3	1.45 (0.66–3.16)	0.353	
	4	2.34 (1.14–4.79)	0.020	
	5	3.87 (1.89–7.94)	2.10 × 10^−4^	
	Continuous ^b^	1.32 (1.13–1.54)	5.17 × 10^−4^	0.60 (0.53–0.67)
Polygenic risk scores (n = 323)				AUROC
	Quintiles	HR 95%CI ^a^	*p*	AUROC (95%CI)
Unweighted, subjects with 80% call rate	1	1.00		
	2	1.27 (0.73–2.21)	0.392	
	3	-	-	
	4	1.85 (1.07–3.19)	0.027	
	5	3.00 (1.75–5.12)	5.90 × 10^−5^	
	Continuous ^b^	1.29 (1.14–1.46)	4.40 × 10^−5^	0.61 (0.54–0.67)
Weighted, subjects with 80% call rate	1	1.00		
	2	1.89 (0.93–3.85)	0.080	
	3	1.64 (0.81–3.32)	0.172	
	4	2.64 (1.37–5.10)	3.80 × 10^−3^	
	5	3.58 (1.85–6.93)	1.50 × 10^−4^	
	Continuous ^b^	1.34 (1.16–1.54)	6.60 × 10^−5^	0.61 (0.55–0.67)

^a^ HR, hazards ratio; CI, confidence interval. All analyses were adjusted for age, sex, and geographic region of origin. ^b^ The unit for the analysis with the continuous variable was the increment of one quintile.

## Data Availability

Genetic data from the CRuCIAL population can be accessed at https://ftp.genyo.es/ (accessed on 28 February 2023) and are available upon reasonable request. Functional data used in this study are available at BBMRI-NL data infrastructure (https://hfgp.bbmri.nl/, accessed on 28 February 2023), where they have been meticulously catalogued and archived using the MOLGENIS open-source platform for scientific data. This allows flexible data querying and download, including sufficiently rich metadata and interfaces for machine processing (R statistics, REST API) using FAIR principles to optimize Findability, Accessibility, Interoperability, and Reusability. These datasets are not publicly available because they contain information that could compromise the research participants’ privacy.

## Supplementary Materials

The supporting information can be downloaded at: https://www.mdpi.com/article/10.3390/ijms24098005/s1.
