# Supplementary material for: Do GWAS-Identified Risk Variants for Chronic Lymphocytic Leukemia Influence Overall Patient Survival and Disease Progression?
Source: Int J Mol Sci. 2023 Apr 28;24(9):8005. doi: 10.3390/ijms24098005 (PMC10178669; doi:10.3390/ijms24098005)
Supplement: Supplementary file 1 [file ijms-24-08005-s001.zip › Cabrera-Serrano_AJ_[1]_Supplementary_Table_S1_List_of_markers.pdf]

Supplementary Table S1. List of GWAS-identified risk variants for CLL.

SNP	Chr.	Nearby gene	Reference Allele	Risk allele	Location	Author (Pubmed ID)
rs4368253	18	*AC107990.1||NFE2L3P1*	T	C	Intergenic	Berndt SI (26956414)
rs58055674	2	*ACOXL*	T	C	Intronic	Berndt SI (26956414)
rs1439287	2	ACOXL	C	T	Intronic	Berndt SI (23770605)
rs7944004	11	*ASCL2. C11orf21*	G	T	Intergenic	Berndt SI (23770605)
rs4987855	18	*BCL2*	A	G	3′ UTR	Berndt SI (23770605)
rs2651823	11	*C11orf21|TSPAN32*	G	A	Intronic	Law PJ (28165464)
rs1476569	4	*CAMK2D*	A	G	Intergenic	Law PJ (28165464)
rs3769825	2	*CASP8*	C	T	Intronic	Berndt SI (23770605)
rs7558911	2	*CFLAR*	G	A	Intronic	Law PJ (28165464)
rs1036935	18	*CXXC1*	G	A	Intergenic	Law PJ (28165464)
rs1359742	9	*DMRTA1*	C	G	Intergenic	Berndt SI (26956414)
rs6546149	2	*DTNB*	C	G	Intronic	Law PJ (28112199)
rs9880772	3	*EOMES|LINC01980*	C	T	Intergenic	Berndt SI (26956414)
rs13015798	2	*FAM126B*	G	A	Intronic	Berndt SI (26956414)
rs6586163	10	*FAS*	C	A	Intronic	Law PJ (28165464)
rs2267708	7	*GPR37*	C	T	Intronic	Law PJ (28165464)
rs35923643	11	*GRAMD1B*	A	G	Intronic	Berndt SI (26956414)
rs2953196	11	*GRAMD1B*	A	G	Intronic	Berndt SI (26956414)
rs3800461	6	*ILRUN*	G	C	Intronic	Law PJ (28165464)
rs9392504	6	*IRF4*	G	A	Intergenic	Law PJ (28165464)
rs391855	16	*IRF8*	T	A	Intergenic	Berndt SI (26956414)
rs898518	4	*LEF1*	C	A	Intronic	Berndt SI (23770605)
rs34676223	1	*MDS2*	A	C	Intergenic	Law PJ (28165464)
rs57214277	4	*MYL12BP2||LINC02363*	C	T	Intergenic	Law PJ (28165464)
rs10936599	3	*MYNN*	T	C	Missense	Speedy HE (24292274)
rs11715604	3	NCK1	A	T	Intronic	Law PJ (28112199)
rs6489882	12	*OAS3*	A	G	Intronic	Law PJ (28165464)
rs140522	22	*ODF3B*	C	T	Upstream	Law PJ (28165464)
rs2236256	6	*OPRM1||IPCEF1*	A	C	Intronic/3′UTR	Speedy HE (24292274)
rs11637565	15	PCAT29|LOC107984788	A	G	Intergenic	Law PJ (28165464)
rs17246404	7	*POT1*	T	C	Non Coding Transcript Variant	Speedy HE (24292274)
rs2511714	8	*POU5F1P2||ODF1*	T	G	Intergenic	Berndt SI (26956414)
rs11083846	19	*PRKD2*	G	A	Intronic	Di Bernardo MC (18758461)
rs888096	2	*QPCT||RNU6-1116P*	G	A	Intergenic	Law PJ (28165464)
rs41271473	1	*RHOU*	A	G	Non Coding Transcript Variant	Law PJ (28165464)
rs73718779	6	*SERPINB6*	G	A	Intronic	Berndt SI (26956414)
rs12638862	3	TERC	G	A	Intergenic	Law PJ (28112199)
rs7705526	5	TERT	C	A	Intronic	Law PJ (28165464)
rs61904987	11	*TMPRSS5||DRD2*	C	T	Intergenic	Law PJ (28165464)
rs926070	6	TSBP1-AS1	G	A	Intronic	Speedy HE (24292274)
rs7254272	19	*ZBTB7A|MAP2K2*	G	A	Intergenic	Law PJ (28165464)
